# Dr. Charles Stedman Bull

**Published:** 1911-05

**Authors:** 


Dr. Charles Stedman Bull, of New York, died suddenly of heart disease April 17, 1911. He was born in New York in 1845, was graduated in arts from Columbia University in 1864, and in medicine from the College of Physicians and Surgeons in 1868. Dr. Bull was one of the foremost ophthalmologists of the country, and at the time of his death was surgeon of the New York Eye and Ear Infirmary, visiting surgeon at the Charity Hospital, professor of ophthalmology at Cornell Medical College, and consulting ophthalmic surgeon at St. Luke's, the Presbyterian, and St. Mary's hospitals. From 1903 to 1907 he was president of the American Ophthalmological Society.

